# Unplanned Excision in Soft Tissue Sarcoma: Current Knowledge and Remaining Gaps

**DOI:** 10.3390/diagnostics15040453

**Published:** 2025-02-13

**Authors:** Tomoki Nakamura, Masahiro Hasegawa

**Affiliations:** Department of Orthopaedic Surgery, Mie Graduate School of Medicine, Tsu City 514-8507, Japan

**Keywords:** unplanned excision, soft tissue sarcoma, magnetic resonance imaging

## Abstract

Soft tissue sarcoma (STS) is a rare and heterogeneous disease, which can result in surgeons not considering STS as a differential diagnosis when they encounter a lump. However, unplanned excision (UE) often occurs in nonspecialized sarcoma centers. Before re-excision (RE) after UE, radiological examinations such as magnetic resonance imaging (MRI) should be performed to determine the surgical margin and conduct a pathological evaluation of the UE. However, differentiating between residual tumor and postsurgical changes remains challenging because of the presence of postoperative edema, hematoma, and seroma on MRI. Propensity score matching analysis showed that patients with STS who underwent RE after UE did not have higher mortality or local recurrence rates than those who underwent planned excision (PE), while RE often requires reconstruction procedures. From the patient’s perspective, one operation (PE) is better than two (UE and RE) because it reduces hospital stays and time away from work. Continuous education about STS is necessary for all surgeons to reduce the incidence of UE.

## 1. Introduction

Soft tissue sarcoma (STS) is a rare and heterogeneous disease that accounts for 0.7% of all kinds of cancers [1]. The age-adjusted incidence of STS was estimated to be 2.4/100,000 people/year in the United States in 2022 [1]. This may explain the high incidence of unplanned excision (UE). While most general surgeons may only encounter a few cases of STS, they may see a lot of small and superficial benign tumors, such as lipomas, epidermal cysts, and schwannomas. Therefore, UE tends to occur in nonspecialized sarcoma centers, where STS is seldom treated [2]. From the data using the bone and soft tissue tumor registry in Japan, the mean incidence of UE for STS was reported to be 11.3% [3]. From the Netherlands Cancer Registry database, 2187 adult patients with STS between 2016 and 2019 who received surgery were registered. Overall, UE accounts for 18.2% of all initial surgeries for STS [2]. Kang et al. reported 553 (36.5%) of 1517 Korean patients with extreme STS who underwent UE using the Health Insurance Review and Assessment Service database over a 4-year study period [4]. Therefore, UE is common worldwide. Efforts should focus on preventing UE and addressing its consequences. We reviewed the relevant literature on the management and clinical outcomes of UE in STS.

## 2. Circumstance of UE in STS

*Unplanned excision* was first described by Giuliano and Eilber [5] and later by Noria et al. [6] to explain tumor resection without preoperative diagnostic devices, such as magnetic resonance imaging (MRI), and without the intent to achieve adequate surgical margins (R0 resection). Due to their relative infrequency, STSs are often not considered during differential diagnoses in many situations. All soft tissue masses should be approached with caution, and the initial assessment should begin with a comprehensive history and physical examination. Clinical features such as larger size (≥5 cm) and rapid growth should raise suspicion for STS [7,8,9], although tumor size of <5 cm that are adherent to the deep fascia or surrounding structures should be treated with suspicion of STS [9]. Current guidelines suggest that any soft tissue mass that is either deep or larger than 5 cm in size should be investigated prior to excision; thus, it is expected that most STSs are resected as UE would be small and superficial. Table 1 shows the literature that includes more than 50 patients who underwent UE over the last 10 years. More than half of the patients had small (<5 cm in size) and superficial STS [9,10,11,12,13,14,15,16,17,18,19,20,21,22,23,24,25,26,27,28,29,30,31].

If preoperative imaging, such as MRI, has been performed, it should be reviewed, as it greatly aids in confirming the accurate site of the primary STS. Furthermore, understanding the imaging appearance of the tumor at presentation is valuable for assessing any potential residual tumor. However, MRI is often not performed in patients who undergo UE. Some authors showed that only 21% and 46% of patients who received UE underwent preoperative MRI [2,22,32,33]. Nakamura et al. reported that MRI was frequently performed by orthopedic surgeons (82%) before UE. In contrast, only half of the patients (48%) who received UE by other surgeons, including plastic, general, and dermatological surgeons, underwent MRI preoperatively. This result suggests that orthopedic surgeons judge the tumor as benign based on MRI reports and proceed with resection, while other surgeons do not suspect the possibility of STS before UE. Even if MRI is performed before surgery, differentiating between STS and benign tumors remains a common challenge in daily clinical practice. Some benign soft tissue tumors, such as lipomas, can be accurately diagnosed using MRI. However, for soft tissue tumors with non-characteristic imaging appearance, MRI is limited in reliably differentiating between benign tumors and STSs [34,35,36,37]. The diagnostic accuracy of standard MRI for distinguishing between malignant and benign soft tissue tumors has been reported to range widely (50–85%) [36]. We recommend that when a nonspecific imaging appearance is observed in a soft tissue mass, the patient should be referred to a sarcoma-specific center for further management, regardless of tumor size.

Even if surgeons suspect STS based on clinical features and MRI examination, another problem with UE is that it is often performed using inappropriate approaches. These include (a) performing the biopsy in line with the longitudinal incision under appropriate anesthesia, avoiding any local injection at the biopsy site; (b) violating only one compartment and avoiding contamination of adjacent joints and neurovascular structures; (c) ensuring careful hemostasis to avoid hematoma formation; and (d) placing drains close to and in line with the surgical incision. Further contamination of the surgical field may necessitate a wide range of surgical margins and require soft-tissue reconstruction at re-excision (RE) [9].

Following UE, standard practice is to perform a new MRI to determine the presence of a residual tumor. However, differentiating between a residual tumor and postsurgical changes remains challenging, as postoperative edema, hematoma, and seroma often confound examinations. The accuracy of MRI depends on the timing of the examination after UE. Four studies involving 50 patients who underwent UE describe the role of MRI in predicting residual tumors. Davies et al. reported that in 104 patients who underwent UE, MRI had a sensitivity of 64% and specificity of 93% for detecting residual tumors [38]. Gingrich et al. reviewed 76 patients with UE and showed that the sensitivity and specificity of MRI for predicting residual tumor cells were 86.7% and 57.9%, respectively, with an overall accuracy of 78.1% [39]. Patkar et al. reviewed 55 patients who underwent oncological scar excision after UE and reported MRI sensitivity and specificity of 86.7% and 90.9 %, respectively, for detecting residual tumors, with an overall accuracy of 88.5%. Nakamura et al. evaluated 97 patients who underwent UE and reported MRI sensitivity and specificity of 97% and 73%, respectively, for detecting residual tumors [22]. A meta-analysis of 10 included studies showed that MRI achieved moderate sensitivity (65.9%) and fairly high specificity (85.1%) [40]. Although new techniques using diffusion-weighted MRI and dynamic contrast-enhanced MRI have been reported for detecting residual tumors [41,42], additional studies are necessary because of the small sample sizes in these studies. Therefore, the limitations of the diagnostic value of MRI should be considered. However, MRI remains a valuable tool before RE after UE. It aids in the planning of the additional resection by identifying the surgical site and UE invasion.

It is essential that the previous UE sample be re-examined at specialized sarcoma centers, because of an error rate of up to 30% in nonspecialist reporting of soft-tissue lesions thought to be sarcomas. Therefore, additional treatment should be considered after the diagnosis of re-review [43].

## 3. Treatment After UE

### 3.1. RE After UE

RE is recommended after most UE with the aim of obtaining complete tumor resection with an appropriately wide margin of resection. The RE area tends to be more extensive during tumor bed excision than during PE because of the need to resect potential areas of contamination, resulting in the need for more reconstructive procedures (flaps and skin grafts) and wider radiation fields. Wang et al. reviewed 148 patients with STS managed with RE and UE (n = 78) and planned excision (PE, n = 70) [21]. The total number of surgeries and reconstruction rates were significantly higher in the UE group than that in the PE group (*p* < 0.001). Furthermore, the average number of reconstructive procedures in patients who underwent UE and RE was three times that in the PE group (*p* < 0.001). Potter et al. reported a higher reconstruction rate (30%) in patients who underwent UE and RE than in those who underwent PE (5%) [44]. Arai et al. analyzed retrospectively 128 patients who underwent PE and 63 patients who underwent UE and RE. They reported a greater number of flaps and grafts in the UE and RE group (71%) than those in the PE group (47%) (*p* = 0.002) [45]. Nakamura et al. created propensity score matching and established a new cohort of 355 patients in each group for both PE and UE, with appropriately balanced baseline covariates. The patients who underwent UE and RE had a higher risk of reconstruction (odds ratio 1.6; 95% confidence interval (CI) 1.180 to 2.180) [9]. Even if the deep-seated STS, Traub et al. reported that the rates of plastic reconstruction and amputation in patents who underwent UE and RE were significantly higher in comparison with the rates for patients who underwent a primary PE [14].

The optimal timing of RE after UE has not been well established except for rhabdomyosarcoma. Liang et al. investigated the effects of delayed RE [17]. The patients were divided into two cohorts: the short-interval group (<30 days) and the long-interval group (≥30 days) according to the median interval. Prognosis did not differ substantially between the two groups. Han et al. reported no difference in outcomes between patients undergoing early RE (before 32 days, median in their series) and those undergoing RE after 32 days [46]. The authors also concluded that there was no difference in disease-specific survival or local recurrence (LR) based on the time from UE to RE. Scoccianti et al. also showed no statistically significant differences in disease-specific survival, LR survival, or event-free survival according to the depth and time from UR to RE (before vs. after 2 months) [11]. Funovics et al. reported that undergoing RE within 12 weeks from initial UE led to a better prognosis [47]. Decanter et al. collected data on 251 patients who underwent UE with R0 or R1 resection, grouping together patients who could have undergone RE but did not intentionally undergo RE and those for whom radiotherapy was chosen because of the high risk of LR [24]. They described 63.8% of the patients were spared from RE after 5 years of follow-up without worsening metastasis-free survival or overall survival (OS), and these patients avoided immediate useless surgery. An advantage of delaying RE until the time of LR is that surgery is better guided by the tumor mass than with immediate RE. Morii et al. compared 45 patients who underwent UE and RE with 32 patients who were managed non-surgically after UE, reporting 5-year local recurrence-free survival (LRFS) of 86.7% (LR: 39 of 45 patients) and 50% (LR: 16 of 32 patients), respectively [48]. RE was an independent predictor of local control in multivariate analysis (*p* = 0.002). Future studies are necessary to identify patients who can benefit from a wait-and-see approach.

Concerning rhabdomyosarcoma, RE should be performed as soon as possible if severe functional loss is not expected. RE has been shown to improve outcome when it can convert IRS Group II or III tumors to IRS Group I when performed before the initiation of adjuvant chemotherapy [49,50]. In pediatric nonrhabdomyosarcoma STS, several authors recommend RE and reported the outcome following RE were similar to that with PE [51,52].

Residual tumors were reported in 19–83% (Table 1). Residual tumor tissue can be a confounding factor influenced by other factors; hence, its presence should be interpreted with caution. Although the existence of residual tumors is thought to reflect the achievement of tumor-free margins, it may also partially reflect tumor invasiveness [44,53].

Patients who received UE have been reported to have worse clinical outcomes than those who received PE; however, some authors have described that outcomes for patients who received UE are similar to or even better than those of patients who received PE. These discrepancies are likely due to differences in characteristics between patients who underwent UE and those who underwent PE. Patients with large and deep STSs are also more likely to be referred to sarcoma-specific centers. In contrast, patients with small and superficial STSs are more likely to undergo UE performed by nonspecialized surgeons.

Recently, some authors have created a group from a study population with balanced baseline characteristics using propensity score matching (PSM) to elucidate the potential influence of UE and PE [9,10,25,30]. Nakamura et al. reported on a cohort of 355 patients who underwent both PE and RE after UE, with appropriately balanced baseline covariates established through PSM. They concluded that patients with STS of the extremities who had RE after UE did not experience worse disease-specific survival (DSS) or LRFS compared to those who underwent PE [9]. Liang et al. created a cohort of 107 patients who underwent both PE and RE after UE, with appropriately balanced baseline covariates established through PSM [17]. Interestingly, the UE and RE group had a longer LRFS (hazard ratio 1.52; 95% CI 1.15 to 2.2), but no difference was observed between the two groups regarding DSS. In a large study, Smolle et al. used an inverse probability-of-UE weight (IPUEW), and time-to-event analyses calculated after IPUEW weighting showed that the UE and RE did not seem to adversely affect prognosis [30]. Without IPUEW, patients who underwent RE and UE had improved OS than patients who underwent PE, whereas the risk of LR was similar. However, after IPUEW, the observed differences in OS disappeared. Charoenlap et al. compared outcomes for patients who underwent UE and RE with those who underwent PE and found that residual disease indicated a worse DSS, although there was very little difference in outcomes when patients were matched by stage [12].

The aim of RE is to acquire clear margins to decrease the risk of LR. The rate of LRFS after RE ranged from 73.5% to 92.8% at 5 years (Table 1). Grimer et al. reported the effects of tumor grade and residual tumors on LR [43]. High-grade STS and residual tumors are frequently associated with LR. Of the patients with high-grade tumors and positive surgical margins after RE, 63% developed LR, whereas only 2% of patients with low-grade tumors and no residual tumor developed LR. Charoenlap et al. reported that residual disease was associated with a worse prognosis for LR and also a higher eventual amputation rate compared to patients who underwent PE (18% vs. 1.8%) [12].

### 3.2. Radiotherapy

The use of radiotherapy (RT) varies across countries, although recent guidelines have emphasized the importance of RT in decreasing the risk of LR. Allignet et al. reported the role of adjuvant RT in patients who underwent UE and RE [15]. The indications for RT were based on the presence of residual disease, surgical margins after RE, and initial adverse criteria: lesion size >5 cm, deep-seated lesion, histologically high-grade, and high-risk histotypes such as epithelioid sarcoma. The median radiation dose was 50 Gy. At 10 years, patients in the RT (n = 108) and no-RT groups (n = 37) showed a cumulative incidence of LR at 10 years of 14.7% and 37.7%, respectively, and a 10 year LRFS of 61.3% and 45.8%, respectively. Kepka et al. reported on 78 patients treated with RT alone after UE. They used a 66 Gy dose and reported a 12% LR rate at 5 years, but with 10 major radiotherapy complications [54]. The reasons for not performing RE, as stated in the medical records, were as follows: risk of severe functional or cosmetic loss (n = 32, 41%); estimated low risk of LR (n = 18, 23%); RE judged as technically difficult (position of scar, skin coverage, patient’s obesity) (n = 15, 20%); patient’s refusal of RE (n = 5, 6%); and unknown reasons (n = 8, 10%). Most patients were treated in the 1970s or the 1980s because they also underwent RE. They concluded that RT is appropriate for patients if severe functional or cosmetic loss may be predicted after RE or they have severe medical conditions.

## 4. Conclusions

UE is common in patients with STS. MRI is useful for setting surgical margins at RE, although its effectiveness is often confounded by the presence of postoperative edema, hematoma, and seroma. RE is the standard treatment of UE. However, the optimal timing of RE after UE remains unclear. Recent studies suggest that a wait-and-see policy is appropriate for select patients who underwent UE. However, further studies are required to clarify these factors. Therefore, radiotherapy instead of RE may be considered if severe functional or cosmetic loss may be predicted after RE or they have severe medical conditions. PSM analysis showed that patients with STS who underwent RE after UE did not experience higher mortality or local failure than those who underwent PE. However, RE often requires reconstruction procedures. From the patient’s perspective, one operation (PE) is better than two (UE and RE), as it reduces hospital stays and time away from work. Continuous education about STS should be necessary for all surgeons to reduce the incidence of UE.

## Figures and Tables

**Table 1:** Clinical characteristics and outcome in patients who underwent unplanned excision.

Authors	Study Cohort	Mean Size	<5 cm (%)	Superficial	RE	Residual Tumor	Recon.	5Y-OS	5-Y DSS	5Y-LEFS	LR
[Ref]	(n)	(cm)	(%)	(%)	(%)	(%)	(%)	(%)	(%)	(%)	(%)
Melis et al.	399		61	84	68						
[2]											
Nakamura et al.	355	4.2	73	59	100		54		90.7	89.7	
[10]											
Broida et al.	87	4.8	65		86	61			72	82	
[11]											
Scoccianti et al.	131		60	57	100	54			93.5	87.6 *	
[12]											
Charoenlap et al.	161	6.2	57	47	100	55	24		79.7	86.9	
[13]											
Traub et al.	94	10.3		0	100	83	47	54		88.3	
[14]											
Gingrich et al.	76	7		21	84	70					
[15]											
Allignent et al.	145		53	41	100	52					
[16]											
Bianchi et al.	103		58	28	100	43					9
[17]											
Liang et al.	129		71	56	100	78			76.6		
[18]											
Shemesh et al.	54	4.9		28	100		26				18.5
[19]											
Traweek et al.	67	3.8		60	100	19	67				
[20]											
Morattel et al.	64	5.6	61	56	100	31					11
[21]											
Wang et al.	78	8.8		23	100	65	40				25.3
[22]											
Nakamura et al.	97	4.2	73	59	85	56	72				
[23]											
Lans et al.	52	3 *		76	60	51	29	83			13
[24]	300		61	48	100			88.4		83	9.3
Decanter et al.	71		68	52	100			87.3		73.5	21.1
[25]	251		47	33	0			88		63.8	31.9
Zaidi et al.	281	4.6		26	100						
[26]											
Nakamura et al.	174	3.2	100	64	89		60			92.8	
[27]											
Alsina et al.	145	6.2			100	81			70.3	84.1	17.9
[28]											
Fromm et al.	156	4.5		36	100	60					8
[29]	29	10.1		3	0						14
Danieli et al.	697	4 *		55	100	27	19.4				8.8
[30]											
Smolle et al.	281		52	50	99		57				15.8
[31]											
Koulaxouzidis et al.	71		52	34	100	54					
[32]											

* Median size. RE; re-excision; Recon., reconstruction; OS, overall survival; DSS, disease-specific survival; LRFS, local recurrence-free survival; LR, local recurrence.

## Data Availability

All data are shown in the present manuscript.
